# Vaccination-Challenge Trials in Beagle Dogs Using Whole-Cell *Leptospira interrogans* Serovar Copenhageni Vaccine: Prevention of Clinical Leptospirosis, Serological, Leptospiremia, Leptospiruria, Cytokines, Hematological, and Pathological Changes

**DOI:** 10.3390/pathogens14070611

**Published:** 2025-06-20

**Authors:** Teola Noel, Rod Suepaul, Abiodun A. Adesiyun

**Affiliations:** 1 School of Veterinary Medicine, Faculty of Medical Sciences, University of the West Indies, St. Augustine 999183, Trinidad and Tobago; teola.noel@sta.uwi.edu (T.N.); rod.suepaul@sta.uwi.edu (R.S.); 2 Department of Production Animal Studies, Faculty of Veterinary Science, University of Pretoria, Private Bag X04, Onderstepoort, Pretoria 0110, South Africa

**Keywords:** bacterin vaccine, effectiveness, clinical signs, cytokines, hematology, pathology

## Abstract

A killed, whole-cell vaccine was produced to induce immunity in dogs against leptospirosis. The vaccine, containing serovar Copenhageni, was produced and administered to 12 beagle dogs at both 8 and 12 weeks of age. Ten unvaccinated dogs of the same age group served as the control group. A live, virulent inoculum of *Leptospira* (1.52 × 10^9^–4.40 × 10^9^ leptospires per dog) was used to challenge the dogs at 2 weeks (Study 1) and 14 months (Study 2) post-booster vaccination. At regular intervals, pre- and post-challenge (PC), the microscopic agglutination test (MAT) was performed to measure antibody titers. Leptospiremia and leptospiruria were determined via culture, and the cytokine, biochemical, and pathological profiles of vaccinates and controls were also assessed. A high antibody response was measurable after booster administration. In Study 1 (onset of immunity), acute leptospirosis was observed in five (100%) out of five unvaccinated dogs. In contrast, no acute clinical leptospirosis developed in vaccinated dogs, except in one (20%) dog with mild clinical signs. In Study 2 (duration of immunity), mild clinical signs were observed in two (40%) of the control dogs, while all vaccinated dogs remained clinically normal. The incidence of leptospiruria and leptospiremia PC was lower in the vaccinated dogs compared to the unvaccinated group. Severe thrombocytopenia occurred in 100% (5/5) of the unvaccinated dogs in Study 1 that exhibited acute severe leptospirosis, whereas 80% (4/5) of the unvaccinated dogs in Study 2 showed mild to moderate thrombocytopenia 3 days after challenge. Four out of five unvaccinated dogs (80%) in Study 1 exhibited icteric tissues and hemorrhages in the lungs and mucosal surfaces of the stomach and intestines. A high IL-10 to TNF-α ratio, observed in the control group of both studies, and severe thrombocytopenia observed in the control group of Study 1, indicative of acute leptospiral disease, were detected. The vaccine prevented acute clinical leptospirosis and reduced the renal carrier state in beagle dogs, and further investigation is required using a larger sample size.

## 1. Introduction

Leptospirosis is a bacterial zoonosis of worldwide importance caused by several species of the pathogenic spirochaete *Leptospira*, comprising more than 200 serovars. The disease has an occupational association, particularly in an agricultural setting. Globally, infection occurs through carrier species. Hence, it remains a significant public health issue in tropical countries, with outbreaks primarily occurring during the rainy season and following floods [1,2,3].

Leptospirosis affects humans and most domestic animals, including dogs, cattle, and pigs. It results in systemic disease manifested by fever, hepatic and renal impairment, and pulmonary and reproductive failure. There is a wide range of clinical signs, although some animals may remain undetected due to infection with serovars that are host-adapted [4].

Leptospirosis has been identified as one of the important bacterial zoonoses that aligns well with the One Health umbrella. The disease in dogs has been suspected of being transmitted to humans in contact with infected dogs [5,6,7,8,9]. Therefore, controlling leptospirosis in dogs is important.

The serovars that cause leptospiral disease in dogs in North America include Canicola, Pomona, Grippotyphosa, Bratislava, Hardjo, Autumnalis, and Icterohemorrhagiae [10,11]. Similarly, Icterohaemorrhagiae, Grippotyphosa, Australis, Sejroe, Bratislava, Hardjo, Autumnalis, Pomona, and Canicola are the main serogroups in Europe to which dogs are exposed [11,12]. In New South Wales, Australia, serovar Copenhageni has been reported as the most common cause of canine leptospirosis [13]. The emergence of new serovars as causes of canine leptospirosis necessitates ongoing epidemiological surveillance and the development of vaccines tailored to cover these emerging serovars [14]. Therefore, for vaccines to be effective in preventing canine leptospirosis, it is essential to monitor the serogroups and serovars prevalent in the region or area to detect any changes that may negatively impact the vaccine’s efficacy against canine leptospirosis [14,15,16,17].

Several commercial vaccines are currently in use globally. The technology used in producing commercial vaccines from the *Leptospira* organism has evolved, including the serovar fraction of a trivalent vaccine, an epitope-based vaccine, and the lipopolysaccharide (LPS) moiety [18,19,20]. Although several commercially available vaccines have been demonstrated to offer protection against clinical leptospirosis and urine shedding of the pathogen in challenged dogs [21], clinical trials on some vaccines have limitations. For example, 84% of individuals are protected against clinical leptospirosis and 88% against renal carriage overall [22,23]. It has been reported that total leptospiral extracts induced complete protection against homologous challenges and partial protection against heterologous challenges. However, LPS fractions protected against homologous but not heterologous challenges, whereas protein extract induced significant protection against both challenge types. Furthermore, protection against clinical disease and carrier status by serovars Canicola, Australis, and Grippotyphosa is inconsistent for some vaccine brands, and such a hurdle is attributed chiefly to methodological difficulties in inducing experimental infection using these serovars [14,21]. Therefore, although cross-protection has been reported to occur against serovars not included in the vaccine panels and against different species of *Listeria* [24], studies by others have suggested otherwise [23,25].

Several cytokines have been demonstrated following bacterial infections, including leptospirosis, some of which perform proinflammatory and anti-inflammatory functions [26,27]. For example, in canine leptospirosis, interleukin-4 is a key component of the complex immune response, potentially contributing to the development of the disease and its severity. However, its exact function is still being researched [28]. Also, it has been reported that interleukin-10 has been suggested to play a complex role in canine leptospirosis, potentially contributing to the disease’s severity and outcome. While it is an anti-inflammatory cytokine, high levels of IL-10 may inhibit the host’s ability to clear *Leptospira* bacteria, leading to chronic carriage effectively. Furthermore, IL-10 deficiency may protect against the disease, suggesting its involvement in its progression [26,29]. TNF-α has been documented to be involved in recruiting neutrophils to the site of infection, contributing to tissue destruction. It directly affects the apoptosis mechanism, activating caspases and the c-Jun NH2-terminal kinase (JNK) and BAX pathways. In severe leptospirosis, TNF-α and IL-10 levels increase, with IL-10 significantly higher in fatal cases [30,31]. IFN-γ plays a crucial role in dogs’ immune response to leptospirosis, particularly in controlling the infection and enhancing the adaptive immune response. It is a key cytokine involved in cellular immunity and plays a crucial role in combating intracellular bacteria, such as *Leptospira* [32].

In leptospirosis, vasculitis commonly occurs through the development of endothelial damage, and inflammatory infiltrates composed of monocytic cells, plasma cells, histiocytes, and neutrophils. The gross pathological changes associated with canine leptospirosis, including petechial and/or ecchymotic hemorrhages, can be widespread, and organs are frequently discolored due to icterus [3,33]. Also, histopathological lesions in canine leptospirosis, which are primarily found in the liver, kidney, heart, and lungs, depend on the disease [3]. Generally, the liver’s structure is not disrupted; however, intrahepatic cholestasis may occur. Hypertrophy and hyperplasia of Kupffer cells are common, and erythrophagocytosis has been reported [34]. Interstitial nephritis is the primary finding in the kidneys, accompanied by intense cellular infiltration composed of neutrophils and monocytes. Leptospires can also be observed within the renal tubules [35,36,37].

In the first documentation of the serovars of *Leptospira interrogans* present in different categories of dogs, including home dogs (vaccinated and non-vaccinated), suspected cases of clinical leptospirosis, serovar Mankarso was detected to be the most frequently detected serologically [38]. Over the years, in Trinidad and Tobago, complaints from numerous veterinarians in small animal practices have led to an increase in the incidence of canine leptospirosis in adequately vaccinated dogs, which has led to the theory that the currently used commercial vaccines that contain serovars Canicola, Icterohaemorrhagiae, Grippotyphosa, and Pomona may be ineffective in Trinidad and Tobago [38]. Although there are common serogroups, it is pertinent to note that the serovars in the commercial vaccines are not the predominant serovars responsible for most clinical leptospirosis cases in Trinidad and Tobago [38]. Therefore, commercially available vaccines may be ineffective in preventing leptospiriosis in dogs in Trinidad and Tobago.

Suepaul et al. [39] reported that local serovars of Copenhageni and Mankarso, both belonging to the serogroup Icterohaemorrhagiae, were the most prevalent serovars in leptospiral infections in dogs and rats. In contrast, serovar Copenhageni was the most commonly isolated serovar from suspected canine cases. This provides further evidence that the vaccines currently used in the country to prevent leptospirosis offer little to no protection to vaccinated dogs due to the serovars they contain. In a follow-up study, Suepaul et al. [40] developed an in-house, killed, whole-cell leptospiral vaccine from locally isolated serovars Copenhageni and Mankarso. They demonstrated that it effectively prevents clinical leptospirosis and renal shedding post-challenge (PC) in a hamster model. Most recently, Arjoonsingh et al. [41] similarly utilized the hamster model and demonstrated that vaccinated and non-vaccinated hamsters challenged with a live virulent strain of *Leptospira icterohaemorrhagiae* Copenhageni exhibited different outcomes. However, in the canine model using beagle dogs, the authors failed to induce clinical leptospirosis in the vaccinated and non-vaccinated dogs, making it difficult to conclude on the efficacy of the whole-cell vaccine.

The current study, therefore, determined the efficacy of a killed whole-cell *Leptospira* vaccine in beagle dogs regarding the prevention of clinical leptospirosis, renal carriage, and shedding in dogs challenged with a higher dosage of the virulent serovar of *Leptospira interrogans*, serovar Copenhageni. The study also compared the manifestation of clinical leptospirosis in terms of onset and duration, as well as the profiles of cytokines, hematology, serum biochemistry, and pathology, between vaccinated and non-vaccinated dogs.

## 2. Materials and Methods

### 2.1. Experimental Design

Two separate vaccination-challenge experiments using beagle dogs were performed to assess the onset of immunity (Study 1) and the duration of immunity (Study 2) by administering an in-house vaccine containing leptospiral serovar Copenhageni. The study design is shown in Table 1.

### 2.2. Dogs Used in the Study

Four beagle dogs, comprising three females and one male, were purchased from a specific pathogen-free (SPF) colony in the USA (Ridglan Farms Inc., Blue Mounds, WI, USA). All dogs used in the study were maintained as an SPF colony, free from exposure to *Leptospira* spp. The dogs were bred, and 23 offspring (male and female puppies) from the breeding stock were used in the study. The dogs were housed in a rodent-free kennel facility with strict biosecurity measures, including placing foot dips at all entry points and using fully covered laboratory wear, gloves, and covered footwear. To ensure that none of the dogs were accidentally exposed to *Leptospira* spp., whether from exposure to reservoir rodent hosts, from the vaccine or challenge inoculum used in the experiments, or from any other source, serological tests were performed to detect any potential exposure before vaccination and challenge administration.

Twenty-two puppies were assigned to two groups using simple random sampling, resulting in a vaccinated and non-vaccinated group (controls). The controls and vaccinated were housed in separate cages at 6 weeks of age, allowing them to acclimate to their new grouping and housing arrangements before baseline sampling began. The control group was handled, treated, and sampled throughout both studies before the vaccinated dogs. Equipment and gear were changed or sterilized between groups to prevent indirect transmission of infection. Proper care and animal welfare were always upheld during the study.

### 2.3. Ethical Approval

Before the commencement of the study, the Research Ethics Committee of the Faculty of Medical Sciences at the University of the West Indies, St. Augustine campus, approved the study protocol, which included the use of animals for experimentation (reference number: CEC-04/05/2011-01). The care and welfare of all animals used in the study were upheld. Additionally, veterinarians at the Veterinary Teaching Hospital of the School of Veterinary Medicine were assigned to provide medical care for the dogs when required, including biannual physical examinations, preventive parasitic control, and pregnancy diagnosis via ultrasonography.

### 2.4. Strain of Leptospira interrogans Used

*Leptospira interrogans* serovar Copenhageni Strain 1S7, which was isolated from a canine clinical leptospirosis case [40,42] and previously used in vaccination-challenge studies involving hamsters and beagle dogs [40,41], was employed in the current study. This strain was used to produce the killed whole-cell vaccine in Studies 1 and 2. For the challenge, a virulent strain of serovar Copenhageni (P 2652, passage 2) was purchased from the Royal Tropical Institute, KIT Biomedical Research, Amsterdam, the Netherlands, since a virulent form of the IS7 could not be attained for this purpose. The virulence of the cultures for the challenge was maintained through passage in 3- to 4-week-old hamsters, as was done by Suepaul et al. [40]. Three passages were conducted to achieve sufficient virulence for the challenge study.

### 2.5. Preparation of Vaccine

Strain 1S7 was grown in liquid Ellinghausen–McCullough–Johnson–Harris (EMJH) medium for 10 to 14 days to achieve a high density of leptospires for vaccine production. The protocol used by Srikram et al. [43] was followed to produce a formalin-killed whole-cell vaccine. Approximately 200–250 mL of live culture was centrifuged at 14,500× *g* for 10 min at 4 °C. The pellet was retrieved and washed in phosphate-buffered saline (PBS) four times. The pellet was then resuspended in 40 mL of 10% neutral buffered formalin for 60 min, followed by four washes with PBS. Aluminum hydroxide and phenol were finally added as adjuvants. The concentration of cells obtained in the vaccine and booster of Study 1 contained 5.3–11.4 × 10^9^ leptospires per mL, while that of Study 2 was 1.4–6.0 × 10^9^ leptospires per mL. The subcutaneous route was used to administer 1 mL of PBS with equivalent additions of vaccine adjuvants to the control dogs.

For both studies, all dogs received two vaccine doses by subcutaneous injection, with the first dose administered at 8 weeks and the second dose administered 4 weeks later, at 12 weeks of age. Vaccine administration and other treatments began in the morning, shortly after the dogs were fed.

### 2.6. Challenge of Dogs with Virulent Leptospires

#### 2.6.1. Study 1

Each dog in Study 1 received 8 mL of a challenge suspension containing 5.5 × 10^8^ leptospires/mL, or 4.4 × 10^9^ leptospires of serovar Copenhageni. The administration included 0.5 mL of the suspension injected subconjunctivally into each eye, and the remaining 7 mL was injected via the intraperitoneal route using a sterile catheter inserted close to the umbilicus.

#### 2.6.2. Study 2

Dogs in Study 2 were similarly challenged, receiving a dose of 1.9 × 10^8^ leptospires/mL or 1.52 × 10^9^ leptospires, administered via the same routes described for Study 1. A 6-week-old beagle dog was added to this study at the time of challenge to demonstrate the virulence of the challenge strain and the dose of *Leptospira* used to produce clinical signs, as it is challenging to induce clinical leptospirosis in adult dogs [44]. This 6-week-old pup was not added as an additional subject to Study 2. Therefore, any data collected from this pup were not pooled with the data collected from the dogs in Study 2 but were analyzed independently. This pup was challenged simultaneously with the dogs in Study 2.

### 2.7. Clinical Assessment of Dogs and Collection of Samples

#### 2.7.1. Clinical Scoring

Dogs in both Studies 1 and 2 were observed daily for 14 days post-challenge (PC) and then once weekly for 5 weeks post-challenge (PC) for signs consistent with clinical leptospirosis, which included depression, anorexia, conjunctivitis, vomiting, diarrhea, jaundice, petechiae, and hematuria. These clinical signs were scored using a standardized protocol recommended by Minke et al. [44]. The dogs were given a score of “0” if the sign was absent and “1” if the clinical sign was present. A sickness score was then calculated using the daily scores for each clinical sign, based on an algorithm that gave triple weighting to the scores for jaundice and hematuria. The sickness score = 1 × (daily score for conjunctivitis/iritis + 1 × (daily score for anorexia) + 1 × (daily score for diarrhea/vomiting) + 1 × (daily score for general appearance) + 3 × (daily score for jaundice) + 3 × (daily score for hematuria). A sickness score of 0 corresponds to no disease, 1–2 to mild disease, 3–4 to moderate disease, and greater than 4 to severe disease. During the clinical assessment, PC, all animals that exhibited severe and irreversible clinical signs with evidence of suffering were humanely euthanized. Personnel who cleaned the canine enclosures and fed the dogs daily were also required to report any clinical manifestations observed in the dogs throughout the study.

#### 2.7.2. Serology

Four mL of whole blood was collected pre- and post-vaccination, including Days −7, 0, 7, and 14 of the first vaccine and Days 0 and 7 of the booster vaccine for both Studies 1 and 2. For Study 2, blood was collected every 2 months after the booster vaccine for 6 months post-vaccination, and then once a month until the challenge. After the challenge, blood was collected once a week for 5 weeks, PC for both studies. The blood was left at 4 °C overnight to clot, and then centrifuged the next day to obtain sufficient serum, which was immediately stored at −70 °C until required.

The sera were used to detect the presence and concentration of anti-leptospiral antibodies by performing the microscopic agglutination test (MAT) in the biohazard safety laboratory, as described by the WHO [45]. The MAT titers were measured for serovar Copenhageni only, since there was no need to include additional serovars for screening purposes. The strain of Copenhageni used in the MAT belonged to a strain of Copenhageni that was also obtained from the Leptospirosis Reference Laboratory in the Netherlands, which is a member of a diagnostic panel of serovars used routinely for quantitative and qualitative MAT for the diagnosis of leptospirosis at the University of the West Indies. MAT titers were expressed as the reciprocal of the highest serum dilution that induced at least 50% agglutination. The Copenhageni serovar used as an antigen for the MAT, designated 8A, was sourced from a panel of *Leptospira* serovars purchased from the Royal Tropical Institute (KIT Biomedical Research), Amsterdam, The Netherlands.

#### 2.7.3. Detection of Leptospires in Blood and Urine

Blood and urine samples were collected once weekly before and after the challenge to detect leptospires in vaccinated and unvaccinated control dogs. To culture leptospires from the unclotted blood, 2 to 3 drops were inoculated into semisolid EMJH medium and incubated at 28–30 °C. The growth of leptospires was assessed visually with the naked eye and microscopically using a dark-field microscope (Olympus Corporation of the Americas, Center Valley, PA, USA) weekly for 8 weeks.

The dogs were administered a diuretic, furosemide, to collect urine samples at 1 mg/kg via intramuscular injection. Approximately 15 min after administering furosemide, aseptically collected free-catch urine samples were obtained from the dogs and placed into sterile sample cups. Immediately after urine collection, two to three drops were inoculated onto semisolid EMJH media and incubated at 28–30 °C, where they were observed for growth, similar to the blood samples.

#### 2.7.4. Cytokine Assay

Four cytokines (interleukin-4 and interleukin-10, TNF-α, and IFN-γ) were assessed in serum samples before and after vaccination and challenge. The cytokines were quantified using commercially available ELISA kits on selected stored sera. A canine TNF-α ELISA development kit and an IFN-γ Do-It-Yourself ELISA were purchased from Kingfisher Biotech, Inc. (St. Paul, MN, USA). Dog IL-10 ELISA kits and IL-4 ELISA kits were purchased from EIAab (Wuhan, China).

#### 2.7.5. Hematology

A complete blood count of each blood sample was analyzed using an IDEXX blood analyzer (IDEXX Laboratories, Westbrook, ME, USA).

#### 2.7.6. Blood Biochemistry

Urea, creatinine, total bilirubin, gamma-glutamyl transferase (GGT), alanine aminotransferase (ALT), creatine kinase, and total protein were measured using the VITROS 4600 Chemistry system machines (Ortho Clinical Diagnostics, Raritan, NJ, USA). These parameters were monitored weekly, both before the challenge and then once a week after the challenge.

### 2.8. Euthanasia

Humane euthanasia was performed by a veterinarian when there was a severe and irreversible disease in the dog, and at the end of the study. The procedure was performed in the laboratory by administering Xylazine (Bomac Laboratories, Manukau City, New Zealand) at a dose of 1.1 mg/kg via the intramuscular route for sedation. After 15 min, pentobarbital natrium (KELA Laboratory, Hoogstraten, Belgium) was administered intravenously at 120 mg/kg.

### 2.9. Post-Mortem Examination

Immediately after euthanasia, the animals were subjected to a gross pathological examination. Samples of kidneys, livers, and lungs were fixed with 10% buffered formalin and processed for histopathological examination following standard procedures. Histological sections were stained with hematoxylin-eosin (HE).

### 2.10. Detection of Leptospires in Organs

Portions of the kidney and liver were aseptically removed from the abdominal cavity after euthanasia. Approximately 1 cm^3^ of each organ was macerated with liquid EMJH medium, which was used to make serial 10-fold dilutions into liquid EMJH medium. The inoculated media were incubated at 30 °C and examined twice weekly for the growth of leptospires [20].

### 2.11. Statistical Analyses

The results of the MAT titer values were expressed as the mean and the standard error of the mean. Analyses for the severity of infection between the controls and vaccinated dogs were done using Fisher’s exact test. The incidence of the type of clinical signs was calculated. The differences in the frequency and duration of being leptospiremic and being a renal shedder were calculated for each group of dogs. Analyses were performed using the Statistical Package for the Social Sciences (SPSS) version 22 using Analysis of Variance (ANOVA)

## 3. Results

### 3.1. Antibody Titers After Vaccination and Challenge

#### 3.1.1. Study 1

**Figure 1:**
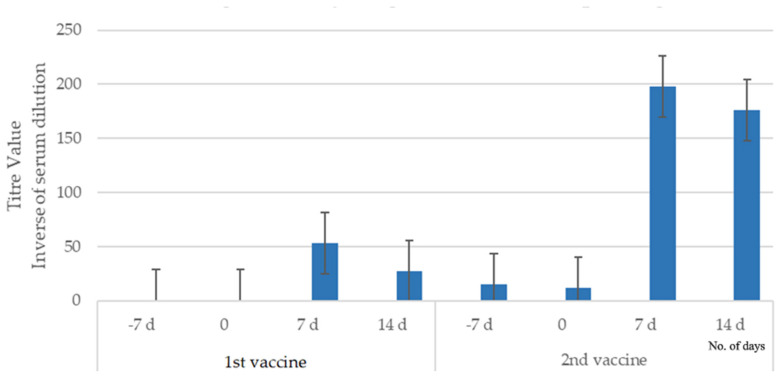
Mean microscopic agglutination titers (±SE) for vaccinated dogs of Study 1 against serovar Copenhageni. The microscopic agglutination test (MAT) measured titers at specific time points, and the mean was calculated for each group. The mean titer values for the controls remained at 0 throughout the vaccination period.

There were no detectable antibodies to *Leptospira interrogans* serovar Copenhageni in the dogs of each study before vaccination began. After the primary vaccination in Study 1, there was an initial increase in MAT mean titers (MT) from 0 to 53, followed by a gradual decrease to 12. Another peak in the mean titer (MT) of 198 was detected 7 days post-booster vaccination, which gradually decreased after 2 weeks but remained considerably high (MT of 176) compared to the post-primary vaccination mean titer (MT of 12), as shown in Figure 1. These differences were statistically significant (*p* < 0.001, Fisher’s exact test).

**Figure 2:**
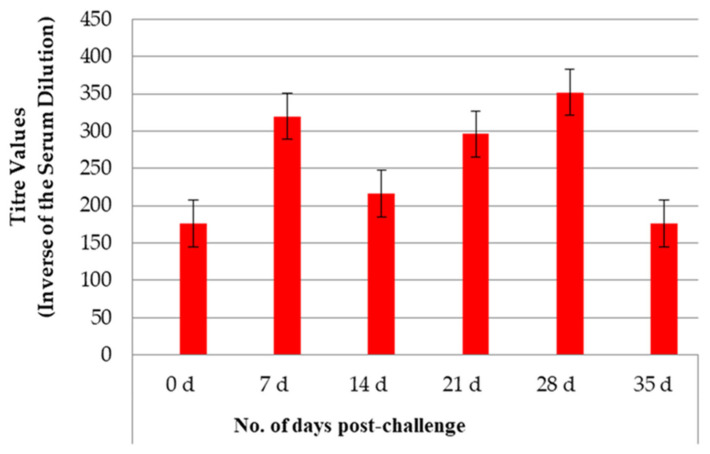
Mean microscopic agglutination titers (±SE) for serovar Copenhageni in vaccinated dogs PC in Study 1. The microscopic agglutination test (MAT) measured titers at specific time points, and the mean was calculated for each group. There are no mean titer values for the controls, as they did not survive after challenge.

MAT titers increased (MT = 320) 7 days PC, then slightly decreased (MT = 216), as shown in Figure 2. The titer levels increased from 21 to 28 days PC and then decreased (MT = 176) by Day 35 PC. MAT titers were not displayed for the control puppies since they did not survive past 7 days PC, the time when MAT titers would be measurable.

#### 3.1.2. Study 2

**Figure 3:**
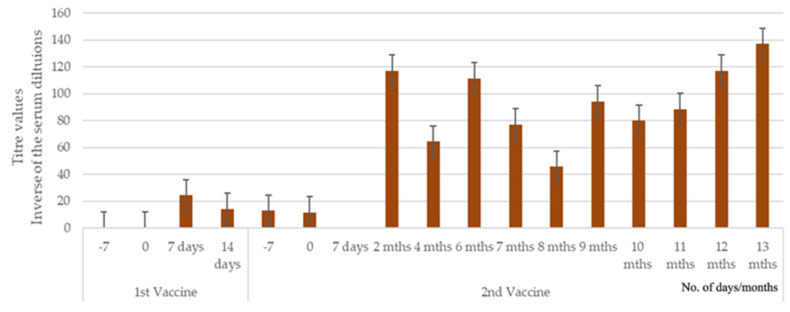
Mean microscopic agglutination titers (±SE) for serovar Copenhageni in vaccinated dogs of Study 2. The antibody titer was measured by the microscopic agglutination test (MAT) at specific time points, and the mean value was calculated for each group. The mean titer values for the controls remained at 0 throughout the vaccination period and measurement.

Figure 3 displays the MAT titers for dogs in Study 2. The MAT titer remained at 0 for the control dogs throughout the pre-challenge sampling period. Following the administration of the first vaccine, there was a slight increase in antibody levels for the vaccinated dogs (MT = 24). Two months after the booster vaccine, and up to the challenge, the mean titers increased to values ranging from 117 to 138. This titer increase between the first and booster vaccines was statistically significant (*p* = 0.004, Fisher’s exact test).

**Figure 4:**
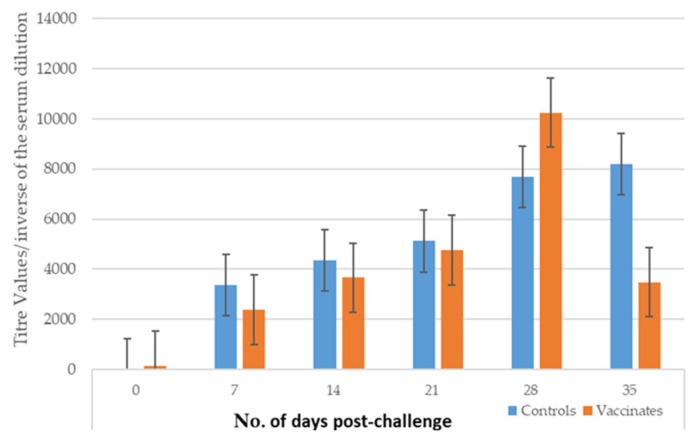
Mean microscopic agglutination titers (±SE) to serovar Copenhageni in dogs PC in Study 2. The MAT measured the antibody titers at specific time points, and the mean found for each group is shown side by side.

The mean titers for the controls were higher than those of the vaccinated dogs at 7, 14, 21, and 35 days PC (Figure 4). On Day 28, the mean titer was higher in the vaccinated dogs (mean titer = 10,240) than in the controls (mean titer = 7680).

### 3.2. Clinical Leptospirosis

#### 3.2.1. Study 1

**Figure 5:**
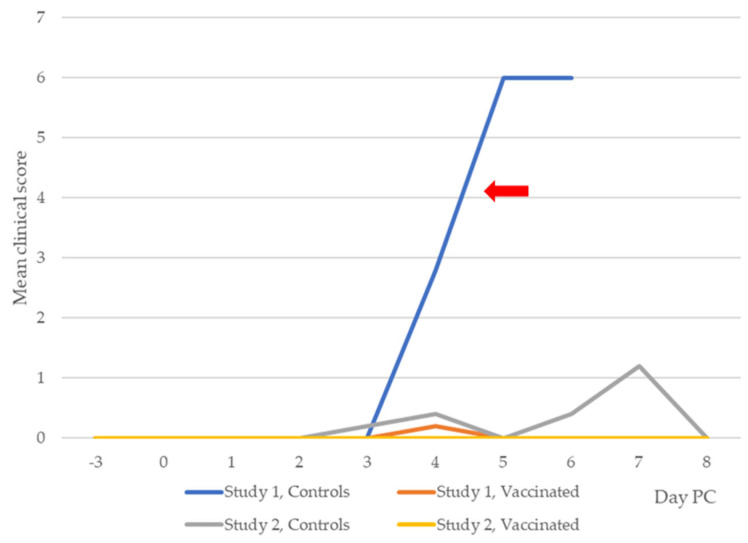
Mean clinical scores recorded in the dogs PC. The mean clinical scores were taken at different time points post-challenge. A score of 0 = no disease, 1–2 = mild disease, 3–4 = moderate disease, and more than 4 = severe disease (indicated by red arrow).

In Study 1, all five control puppies exhibited clinical signs of leptospirosis, which included depression, inappetence, conjunctivitis, vomiting, bloody diarrhea, dehydration, and jaundice within 4 to 5 days PC. In keeping with ethical guidelines, all puppies were humanely euthanized after exhibiting severe clinical signs. However, none of the five vaccinated puppies exhibited severe clinical signs, with only one showing a mild clinical sign of conjunctivitis. The conjunctivitis disappeared without any therapeutic intervention within 24 h. The differences in the frequency of exhibited clinical signs between the control and vaccinated puppies were statistically significant (*p* < 0.001, Fisher’s exact test). The clinical scores of the dogs in Studies 1 and 2 are displayed in Figure 5. When severe disease was attained (red arrow), humane euthanasia was performed. Severe disease occurred when the clinical score was 4 or higher.

#### 3.2.2. Study 2

In Study 2, only two out of the five control dogs displayed mild clinical manifestations, specifically depression, anorexia, and conjunctivitis, which were quickly resolved within 4 days. Among the seven vaccinated, no evidence of clinical leptospirosis was observed during the study period. The 6-week-old, unvaccinated puppy, which was challenged with serovar Copenhageni, developed severe clinical leptospirosis within 4 days of PC. This finding demonstrated that the strain of serovar Copenhageni used for the challenge was virulent since it induced clinical leptospirosis in the puppy. The vaccine prevented clinical leptospirosis in all (100%) seven vaccinated dogs, compared with 3 (60%) of the five unvaccinated controls, which did not exhibit clinical signs of leptospirosis PC. Table 2 shows the incidence of clinical leptospirosis in control and vaccinated dogs of both studies.

### 3.3. Leptospiremia

#### 3.3.1. Study 1

**Figure 6:**
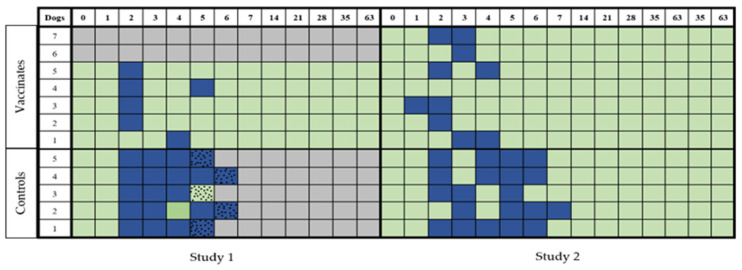
Detection of leptospiremia in dogs from Studies 1 and 2. The presence of leptospires in blood PC was measured by culturing the blood in EMJH medium for leptospiral growth. Positive samples (blue squares) and negative samples (green squares) were recorded for each dog in the two groups (vaccinated and unvaccinated) for both studies. When severe clinical disease was recorded (speckled squares), humane euthanasia was performed. Gray squares indicate that no samples were collected.

All vaccinated dogs developed leptospiremia for one to two days, with the first positive blood sample detected on Day 2. Leptospiremia was detected in all the controls for at least four days, and it persisted for up to five days in some dogs (Figure 6).

#### 3.3.2. Study 2

All control dogs exhibited leptospiremia for 3 to 5 days, while six out of seven vaccinated dogs developed leptospiremia for 1 to 2 days (Figure 6). Leptospiremia was detected in the 6-week-old puppy for two consecutive days, beginning from Day 2 PC.

Overall, 42.1% (40/95) of the samples collected from control dogs of both studies were positive for leptospiremia, while 9.5% (15/158) of vaccinated dogs were positive for leptospiremia. The difference was statistically significant (*p* < 0.0001).

### 3.4. Leptospiruria

#### 3.4.1. Study 1

Among the five vaccinated dogs, shedding was detected at least once to three times on Day 3. Four out of five dogs experienced leptospiruria as late as 63 days PC. Among the five control dogs, leptospirosis was detected as early as Day 3 and as late as Day 5 in two dogs.

Since more samples were collected from the vaccinated group (as all dogs survived the colonization challenge), a longer timeline allowed for renal migration and colonization, and leptospirosis was detected at a higher (22.2%) rate compared to the unvaccinated dogs (16.7%).

**Figure 7:**
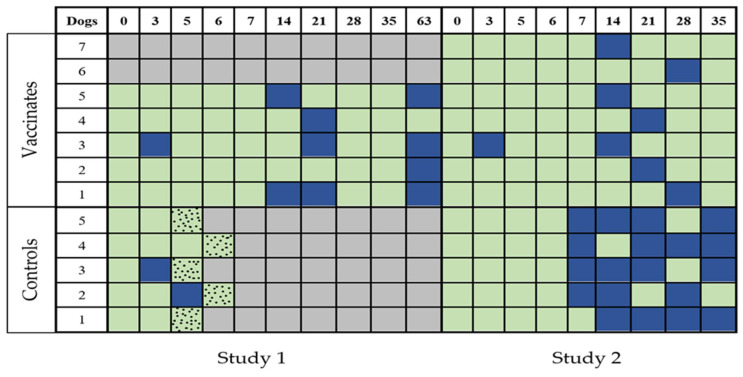
Detection of leptospiruria in dogs from Studies 1 and 2. The presence of leptospires in urine PC was measured by culturing the urine in EMJH medium for leptospiral growth. Positive samples (blue squares) and negative samples (green squares) were recorded for each dog in the two groups (vaccinated and unvaccinated) for both studies. When severe clinical disease was recorded (speckled squares), humane euthanasia was performed. Gray squares indicate that no samples were to be collected.

Figure 7 illustrates the individual leptospiruria results for the dogs in Studies 1 and 2. The figure represents the detection of leptospiruria, indicating the presence of leptospirosis (urine shedding) in dogs from both studies.

#### 3.4.2. Study 2

The seven vaccinated dogs shed leptospires in the urine on day 3 PC. They were positive for leptospirosis for 1–2 sampling days. Among the five control dogs, leptospirosis was detected first on Day 7. All dogs were positive until Day 35, for 3–4 sampling days.

The overall frequency of shedding is higher in the controls, at 33.9% (21/62), compared to the vaccinated, at 15.9% (18/113). The difference was statistically significant (*p* = 0.0064).

### 3.5. Cytokines

#### 3.5.1. Tumor Necrosis Factor-Alpha (TNF-α)

**Figure 8:**
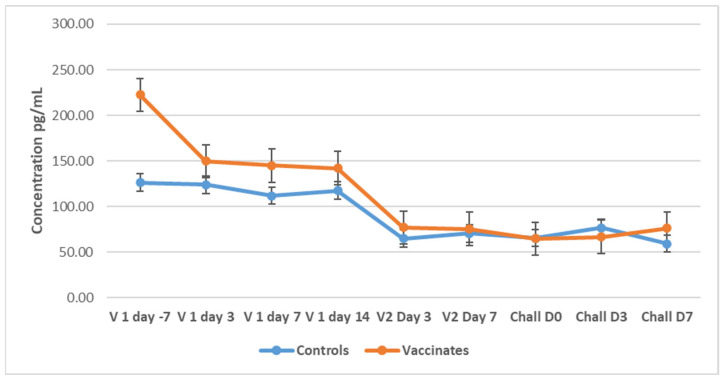
Mean TNF-α (±SE) concentrations for control and vaccinated dogs in Study 1. The mean TNF-α levels were calculated for the dogs of Study 1 (n = 10) at different time points during vaccination and challenge. TNF-α was measured using a canine TNF-α ELISA development kit from Kingfisher Biotech, Inc. (St. Paul, MN, USA).

In Study 1, baseline concentrations of TNF-α were at high levels, a mean of 126.35 pg/mL (range: 114.00–146.00 pg/mL) in control dogs, and a mean of 222.40 pg/mL (range: 114.00–359.00 pg/mL) in vaccinated dogs before administration of the first vaccine Figure 8). There was a gradual decrease in the levels in both groups of dogs until Day 14. On Day 3 after the administration of the booster vaccine, there was a sharp decline by 50% in the previously measured level of TNF-α in both groups, which remained relatively constant until one week PC. Generally, the vaccinated group of dogs had a higher mean concentration of TNF-α than the control group throughout Study 1 (P = 0.000) (Friedman’s two-way analysis of variance).

**Figure 9:**
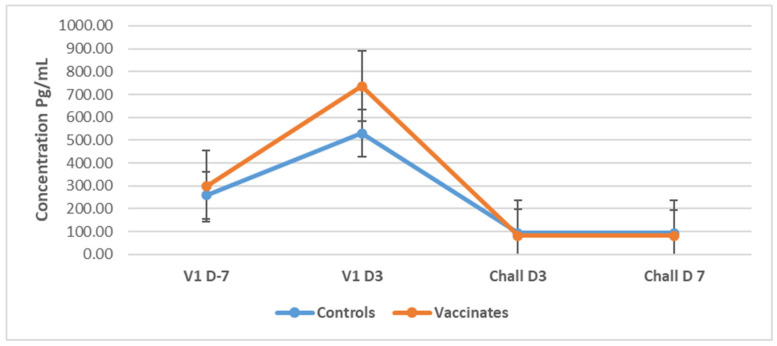
Mean TNF-α (±SE) concentrations for control and vaccinated dogs in Study 2. The mean TNF-α levels were calculated for the dogs of Study 2 (n = 12) at different time points during vaccination and challenge. TNF-α was measured using a canine TNF-α ELISA development kit from King Fisher Biotech, Inc. (St Paul, MN, USA).

In Study 2, there was an increase in circulating TNF-α concentration in both control and vaccinated dogs 3 days after the first vaccine (Figure 9). This increase was more pronounced in the vaccinated dogs than in the control group. Levels were steadily low and constant from 3 days after both groups of dogs were challenged. No significant changes were observed as a result of the challenge.

#### 3.5.2. Interleukin-4 (IL-4)

No significant changes were observed in IL-4 levels from the second vaccine administration to the post-challenge period.

#### 3.5.3. Interleukin-10 (IL-10)

Mean IL-10 concentrations remained relatively constant and similar for the control and vaccinated dogs. The baseline mean concentrations were 136.80 pg/mL for the control dogs and 114.50 pg/mL for the vaccinated dogs. The mean concentration of IL-10 remained steady until 4 days after challenge, where there was a significant increase (*p* = 0.000) in the control dogs, which reached the highest mean concentration of 311.67 pg/mL on Day 6 PC. However, this dramatic increase in mean IL-10 concentration between Days 4 and 6 PC was not detected in vaccinated dogs, where a slight decrease in concentration was detected instead (Supplementary Data, Figure S1).

In Study 2, IL-10 levels showed a similar pattern of production when both groups of dogs were compared. However, there was an increase in IL-10 concentration in the control dogs 3 days PC. The increase in the mean concentration of IL-10 continued to reach 253.75 pg/mL on Day 7 PC when the last sample collected was tested. In the vaccinated dogs, however, a mild, gradual decrease in mean IL-10 concentration was observed (Supplementary Data, Figure S2).

#### 3.5.4. Interferon-Gamma (IFN-γ)

There were no significant differences in IFN-γ concentrations between the control and vaccinated groups of dogs in Study 1. Mean concentrations remained at approximately 25 pg/mL throughout the vaccination and challenge stages of the study. In Study 2, the mean baseline levels of IFN-γ for the controls (51.75 pg/mL) were approximately twice the level quantified for the vaccinated dogs (27.38 pg/mL). From subsequent measurements, no significant differences were detected within the controls (mean range of 26.75 pg/mL to 29.15 pg/mL) and the vaccinated dogs (mean range of 28.25 pg/mL to 33.83 pg/mL).

### 3.6. Hematology

#### 3.6.1. Platelets

**Figure 10:**
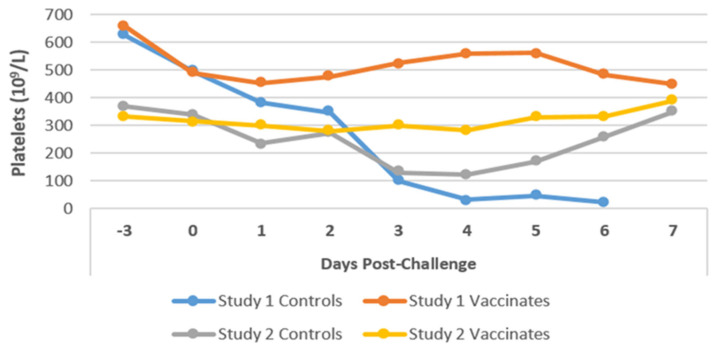
Mean platelet levels in the dogs of Studies 1 and 2. The mean platelet levels were measured for dogs of Study 1 (n = 10) and Study 2 (n = 12) at different timepoints post-challenge. Platelets were measured using an IDEXX blood-analyzing machine (IDEXX Laboratories, ME, USA).

The mean platelet counts remained fairly constant and within normal levels (range 12–19 × 10^9^/L) throughout the post-administration period for both groups of dogs in Studies 1 and 2, following the first and second vaccine administrations (Figure 10). However, PC, moderate to severe thrombocytopenia (mean value of 100 × 10^9^/L) was observed in the control group of dogs as early as Day 3 PC in all five dogs of Study 1, which continued to decrease daily until the animals were euthanized due to the onset of severe clinical disease. The last mean value recorded, on Day 6 PC, was 22 × 10^9^/L.

Similarly, in Study 2, the mean platelet count fell below baseline levels from Day 3 PC in four out of the five control dogs. This mild to moderate thrombocytopenia (130.60 × 10^9^/L) remained within this range for three consecutive days (130.6 × 10^9^/L on Day 3, 122.60 × 10^9^/L on Day 4, 169.80 × 10^9^/L on Day 5), after which the platelet count increased to reach the normal limits. The platelet levels for the vaccinated dogs remained within the normal limits.

Changes in platelet levels are shown from 3 days before the challenge to 1 week post-challenge.

#### 3.6.2. White Blood Cell Counts

In Study 1, control dogs’ mean white blood cell (WBC) counts increased as early as Day 1 PC (Supplementary Data, Figure S3). On Day 3, it was noted that the mean WBC counts dropped below baseline levels when measured on that day. A leucocytosis was observed between Days 4 and 6 PC (21.54–28.21 × 10^9^/L). The mean WBC counts in the vaccinated dogs remained relatively constant; however, a mild increase from baseline values was observed on Day 1 PC, similar to the control dogs (20.33 × 10^9^/L).

In Study 2, leucocytosis was observed on Day 1 for both groups of dogs, similar to the findings in Study 1. However, the vaccinated group had a higher mean WBC count per mL than the control dogs (controls = 19.58 × 10^9^/L, vaccinates = 27.63 × 10^9^/L). From Day 2, mean WBC counts fell to normal limits and remained unchanged. In both studies, a mild neutrophilia was observed on Day 1 post-vaccination (PC) for both controls and vaccinates, which returned to normal limits thereafter. The vaccinated group was noted to have a higher level of neutrophilia than the controls on Day 1 PC: Vaccinates: 18.52 × 10^9^/L (Study 1) and 23.50 × 10^9^/L (Study 2) compared with control dogs: 18.01 × 10^9^/L (Study 1), and 17.52 × 10^9^/L (Study 2).

#### 3.6.3. Biochemistry

##### Alanine Transaminase (ALT)

There was a sharp increase (mean value of 120.60 U/L) from baseline levels (69.40–81.40 U/L) in alanine transaminase (ALT) on Day 5 PC for all control dogs. In the vaccinated group, only one dog had a high ALT level (243.00 U/L) on Day 5 PC, which subsequently decreased to normal (72.00 U/L) on Day 7 PC (Supplemental Data, Figure S4).

##### Bilirubin

Mean bilirubin values were above the upper limit of the reference range (reference range of values less than 5.13 µmol/L) On Days 3 and 5, PC levels in the control dogs, the bilirubin values were 9.23 µmol/L and 7.52 µmol/L, respectively, while bilirubin values in the vaccinated dogs remained within normal limits.

##### Urea and Creatinine

Mean values for urea and creatinine were above the upper limit of the reference range (urea: 1.8–3.9 mmol/L, creatinine: 35–44 µmol/L) for the control dogs on Days 1 (urea = 5.67 mmol/L, creatinine = 54.62 µmol/L), 3 (urea = 5.04 mmol/L, creatinine = 45.81 µmol/L) and 5 (urea = 4.07 mmol/L, creatinine = 51.10 µmol/L). All changes in the mean urea and creatinine levels. The values for vaccinated were within the normal mean ranges for both parameters.

##### Serum Protein

Mean serum protein levels just exceeded the upper limit of the reference range. A 43–49 g/L range was observed for the control dogs on Days 3 and 5, while PC levels were 53.20 g/L and 55.80 g/L, respectively, in the vaccinated group. Mean serum globulin levels were high for the control dogs on Days 3 and 5 PC, 25.40 g/L and 24.60 g/L, respectively, for a reference range of 18 to 23 g/L, while mean levels in the vaccinated group of dogs were high from Days 3 to 35 PC (range 23.40–28.20 g/L). Only on Day 7 PC were the globulin levels within normal limits. Gamma-glutamyl transferase (GGT) levels were within normal limits for all dogs in Studies 1 and 2.

### 3.7. Pathology

#### 3.7.1. Gross Pathological Findings

**Figure 11:**
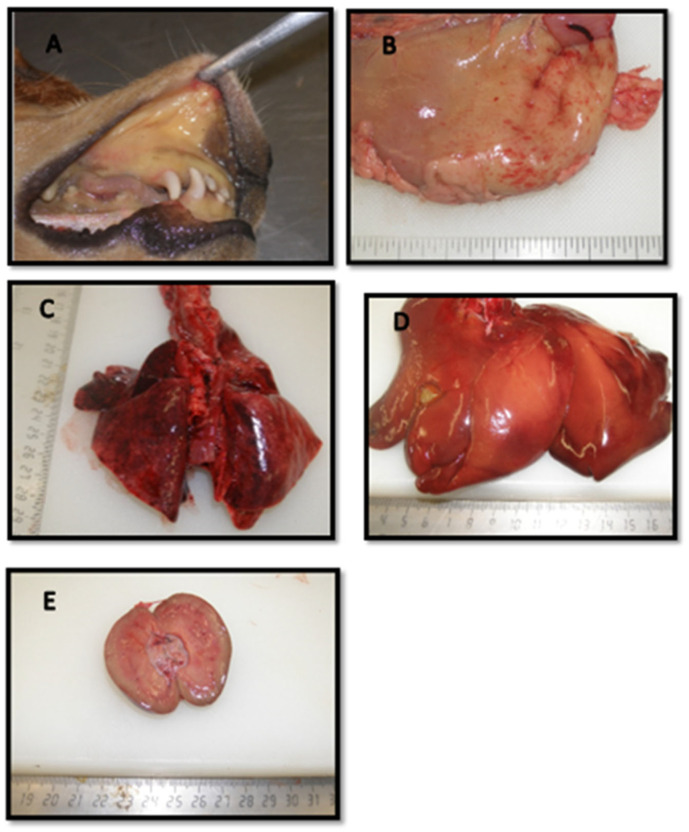
Post-mortem examination of unvaccinated dogs (n = 5) of Study 1. (**A**) Icteric oral and mucous membranes. (**B**) Petechiae and ecchymoses on the serosal surface of the stomach. (**C**) Patchy congestion and/or hemorrhage throughout the lungs. (**D**) Slightly enlarged liver with rounded edges. (**E**) Edematous kidney that oozed clear fluid on cutting.

In all five control dogs in Study 1, significant post-mortem lesions consistent with leptospiral infection were observed. These dogs showed severe, irreversible clinical disease before being humanely euthanized and thereafter subjected to gross pathological examination (Figure 11). Four out of five dogs (80%) showed icteric tissues, pulmonary hemorrhage, and hemorrhages on the mucosal surfaces of the stomach and intestines. Hemorrhages on the serosal surfaces of the stomach and/or intestines were detected in three dogs (60%). Edematous kidneys were also found in three out of the five control dogs (60%). Hepatic changes, indicated by an enlarged liver with rounded edges, were detected in one (20%) control dog in Study 1.

Post-mortem examinations were conducted on all dogs in Study 2. However, no gross pathological lesions were detected in vaccinated or non-vaccinated (control) dogs. Organ sections were subsequently collected for histopathological examination.

#### 3.7.2. Histopathological Findings in Dogs in Study 2

**Figure 12:**
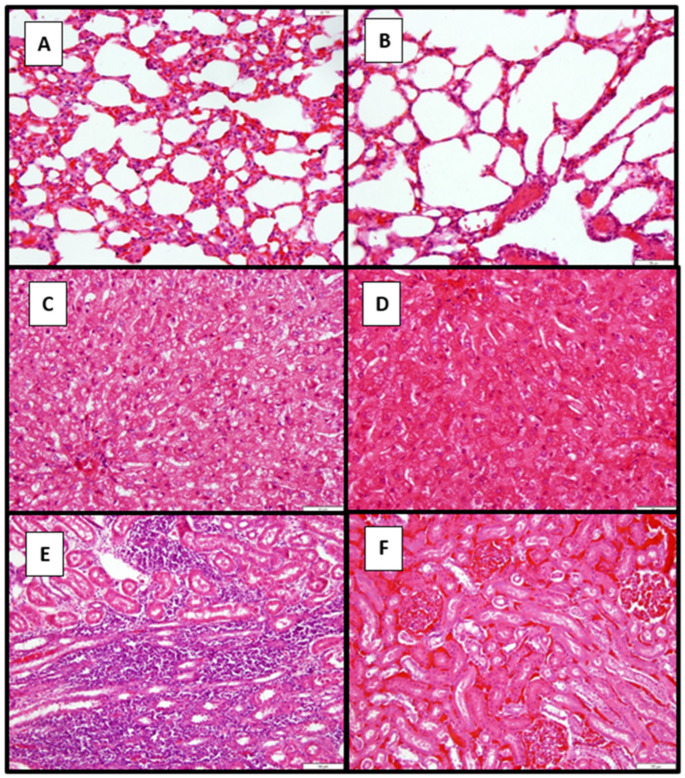
Light micrographs (hematoxylin and eosin) of dogs’ lung, liver, and kidney sections (controls and vaccinates) of Study 2. (**A**) The lung of an unvaccinated dog showing mild interstitial expansion of pulmonary tissue. (**B**) The lung of a vaccinated dog is showing normal lung architecture. (**C**) The liver of an unvaccinated dog is showing some cytoplasmic vacuolation of hepatocytes. (**D**) The liver of a vaccinated dog is showing normal hepatic tissue. (**E**) The kidney of an unvaccinated dog is showing interstitial cellular infiltration. (**F**) The kidney of the vaccinated dog is showing normal kidney architecture.

In Study 2, where no gross pathological lesions were detected in all vaccinated or unvaccinated controls, organ samples of seven dogs were subjected to histological examination (Figure 12). These dogs comprised three of the five control and four vaccinated dogs. In the control dogs, there was a mild diffuse interstitial expansion of pulmonary tissue, with lymphocytes and macrophages present in the lungs of two dogs (A) compared to the normal architecture of a vaccinated dog (B). The sections of the lung tissues from three dogs showed no abnormalities, except for one, which exhibited mild to moderate interstitial expansion with lymphocytes and macrophages upon examination.

Changes in the liver included cytoplasmic vacuolation of hepatocytes throughout the parenchyma (C) as compared to a vaccinated dog with similar histological changes, but to a lesser degree (D).

Vesicular cytoplasm within the epithelium of cortical tubules was observed in approximately 30% of the kidneys. There were multiple small interstitial infiltrates of plasma cells, lymphocytes, and some macrophages in the cortex (predominantly at the cortico-medullary junction) in one of the kidneys examined (E), whereas the renal cortex of the vaccinated dog (F) showed minimal to no cellular infiltration. Three out of four dog kidney sections revealed mild vesicular cytoplasm of the cortical tubular epithelium; while one dog showed no histological abnormalities, the other did.

The small intestine sections revealed mild surface and crypt epithelial hyperplasia in the three control dogs, showing mild to moderate lymphoid area expansion. The changes detected in the small intestine of the four vaccinated dogs were similar to those observed in the control dogs.

### 3.8. Isolation of Leptospires from Organs

The inoculated media of liver and kidney homogenates from dogs in Studies 1 and 2 were all negative for leptospiral growth.

## 4. Discussion

The MAT titer response generated from Studies 1 and 2 after primary and secondary vaccinations is significant, showing increases 7 days after each vaccination, with a more substantial response observed after the second vaccine was administered compared with the first. This represents the humoral response by the immune system upon exposure to the antigens in the vaccine [46]. Immunity produced by bacterins can produce an IgM antibody response with little memory response [15,47]. However, a long-term memory response was observed in Study 2, indicating the presence of high titers up to 14 months after the booster. The post-booster MAT titers remained high, as the vaccine and booster concentrations were high. This would stimulate a large production of IgG antibodies, resulting in a high titer concentration for a prolonged duration, as illustrated in Study 2. However, this contrasts with other vaccination studies [41,48,49].

It is essential to note that all dogs were seronegative before vaccination, and the control group remained seronegative during the post-booster period. This provides evidence that other sources of leptospiral infection did not occur in the dogs at this time, resulting in the high PC antibody titer response. PC and MAT titers were higher in the unvaccinated dogs than in the vaccinated group, a finding similar to that reported in other similar studies [41,44,48].

All the unvaccinated controls in Study 1 developed acute leptospirosis within 7 days PC before they were humanely euthanized. The severe nature of the disease can be attributed to the high dose, a common finding in young dogs, and the use of a virulent challenge strain. In agreement with our study, Minke et al. [44] reported that seven of eight unvaccinated puppies challenged with serovar Canicola developed severe disease. In their second study, using serovar Icterohaemorrhagiae, the authors reported severe leptospirosis in 6 of 10 unvaccinated dogs after challenge. These findings can demonstrate the differences in the severity of clinical disease induced by different serovars of *Leptospira* spp. reflecting their relative virulence. The fact that the strain of serovar Copenhageni used in the current study induced severe leptospirosis in all unvaccinated puppies is evidence that it is highly virulent. On the contrary, Arjoornsingh et al. [41], using the same locally isolated serovar, Copenhageni 1S7 employed in the current study, failed to induce clinical leptospirosis with two doses (1–2.5 × 10^8^ and 1–2.5 × 10^8^ per mL) in beagle dogs in two trials. The authors attributed the failure of both doses to elicit clinical leptospirosis to the loss in virulence following several laboratory subcultures to maintain the organism; the virulence of the bacteria would have been reduced or lost, as suggested by others [50,51,52]. Therefore, in the current study, although the same local isolate of serovar Copenhageni 1S7 was used for vaccine production in both studies, a virulent strain of Copenhageni obtained from a reference laboratory (the Royal Tropical Institute, KIT Biomedical Research, Amsterdam) used at a higher dose (5.5 × 10^8^ per mL) induced clinical leptospirosis in the puppies. Another factor that facilitated the manifestation of clinical signs in the puppies’ challenge was the use of a high-challenge dose of leptospires, which was in a similar range to that used by Minke et al. [44].

The detection of leptospires in the blood of all dogs (vaccinated and controls) PC demonstrates the migration of leptospires to the circulatory system, which occurred from 2 days PC in most dogs of both studies, as expected [15,41]. Leptospires were isolated at a significantly lower frequency from vaccinated dogs (9.5%) compared to control dogs (42.1%) within the first 7 days PC, and the duration of leptospiremia was considerably shorter in vaccinated dogs than in control dogs. These findings suggest that the vaccine may decrease the duration of leptospires circulating in the blood after infection. Vaccination with a leptospiral vaccine in dogs helps reduce the frequency and duration of leptospiremia by stimulating the immune system to recognize and neutralize *Leptospira* spp. before systemic infection can be established. The vaccine induces antibody production that targets pathogenic *Leptospira* serovars, preventing bacterial replication and dissemination in the bloodstream [53,54]. According to Minke et al. [44], leptospiremia was reported in control dogs after challenge with serovars Canicola and Icterohaemorrhagiae, which lasted 10 and 6 days, respectively. However, the authors reported that the vaccinated puppies were negative for leptospiremia PC. In the same study, leptospiremia was detected in the vaccinated dogs of the long-term study (challenged 14 months after booster vaccination), at a lower frequency and shorter duration than in control dogs. Klaasen et al. [48] also detected leptospiremia in vaccinated dogs at a lower frequency and shorter duration than in non-vaccinated dogs. The findings reported in both studies align with the data obtained in our Study 2 regarding duration.

All dogs in both studies shed leptospires in their urine in the current study. Therefore, the renal carrier state in the dogs persisted. However, similar to the pattern observed for leptospiremia, the duration and frequency of shedding were significantly lower in vaccinated dogs than in unvaccinated control dogs. Others reported similar findings, where leptospiruria occurred in vaccinated dogs challenged with serovar Canicola [41,44,48]. However, our findings showed that shedding happened at a lower frequency and duration than in unvaccinated dogs. Vaccination against leptospirosis in dogs is crucial in reducing the frequency and duration of leptospiruria. It produces a robust humoral response that reduces renal colonization of *Leptospira* serovars and urinary shedding [21,55]. Our study, however, is at variance with published reports by others, which have detected no renal shedding in PC with serovar Icterohaemorrhagiae [44,48], Copenhageni [41], Grippotyphosa, Australis, and Canicola [56] in vaccinated dogs. The differences in research findings may again be attributed to the immunogenicity of different serovars of *Leptospira* spp. Additionally, the discrepancies between the reported studies and the current research may be attributed to the immunogenicity of different serovars of *Leptospira* spp. and the varying doses of vaccination and challenge inoculum used in these studies.

Notably, in our Studies 1 and 2, shedding occurred as early as 3 days PC, contrary to published reports where shedding was first detected after 16 days [44], and 6 days PC [48] The early detection of renal shedding in the vaccinated dogs in the current study may be attributed to mild injury of the cortical tubular epithelium of the kidneys that may have occurred in the dogs. Similar vaccination-challenge studies used high-challenge doses, ranging from 5 to 56 × 10^8^ leptospires [44] and 5 to 20 × 10^8^ leptospires [48], where they also detected renal shedding in some of their vaccinated dogs. A high challenge dose may also be attributed to the early leptospiremia resulting from a high leptospiral load, which allows some leptospires to surpass the host’s immune defenses. However, Klaasen et al. [56] were able to prevent renal shedding in their vaccinated dogs, even at a high challenge dose (5–100 × 10^8^ leptospires), using serovars Canicola, Icterohaemorrhagiae, Grippotyphosa, Australis, and Copenhageni for the challenge. Similarly, Arjoonsingh et al. [41] did not record leptospiruria in the vaccinated dogs of their study, which used a challenge inoculum of 2.5–62.5 × 10^8^ leptospires/mL of the Copenhageni strain IS7, the same strain used for vaccination in this study. The lack of renal shedding in vaccinated dogs in this study may be due to the vaccine’s protective effects that prevent renal colonization. However, it can also be due to the lack of a virulent mechanism present in the Copenhageni serovar used in the study. Non-virulent strains will lack virulent factors that allow colonization and persistence in the renal tubules [4].

It is worth noting that the vaccine produced in the current study using serovar Copenhageni prevented clinical leptospirosis and reduced the shedding of the pathogen in challenged dogs. This is because the serovar has been documented to be, along with serovar Mankarso, predominantly circulating in dogs and rats in the country [38,39,42]. Additionally, there are reports of cross-protection within the serogroup for serovars Pomona, Autumnalis, and Icterohemorrhagie [23,57] and Copenhageni [49]. However, there is also evidence of no cross-protection or reactivity only within specific serogroups and serovars [58] and very little with heterologous groups of *Leptospira* spp., including Copenhageni and Canicola [59]. Suepaul et al. [40] also demonstrated little to no cross-protection of the commercially used canine vaccine in the hamster vaccine-challenge model using locally isolated serovars, specifically the Copenhageni and Mankarso strains. It was also demonstrated that the killed whole-cell vaccine produced from a locally isolated serovar Copenhageni prevented clinical leptospirosis and renal shedding in a hamster model in the country. In contrast, the most commonly available vaccine did not [42]. The cross-protectivity component of the vaccine could not be assessed in our study because only one serovar (Copenhageni) was used.

Since leptospiral LPS is recognized primarily by Toll-like receptor 2 (TLR 2), it was expected to see a rise in the concentration of TNF-α after challenge of the dogs of both studies, since an administration of live virulent leptospires will produce an acute, inflammatory response by the body, which was recorded in the current study. Compared to data generated by Vernel-Pauillac et al. [60], who measured the gene expression levels of specific cytokines in hamsters challenged with leptospires, they reported a high expression level from 2 h after challenge. However, the authors reported that TNF-α expression levels quickly declined from 4 h after challenge, remaining relatively low for the few days measured. Considering that the dogs in both Studies 1 and 2 were sampled 24 h post-challenge (PC), the peak levels of TNF-α would have already passed and therefore not been detected. This pattern of early disappearance of the cytokine was also reported by Oliver et al. [61], who observed peak concentrations of TNF-α levels two hours after endotoxin administration in an in vitro whole blood model, which then rapidly declined, with a measured half-life of 18.2 min. High TNF-α levels were also detected in the study by Arjoonsingh et al. [41] 7 days PC. Similarly, high TNF-α expression levels in the hamster were observed from 1 to 22 h post-infection, returning to baseline levels from 24 h post-infection [62].

The post-challenge response of high IL-10 concentrations in both studies’ control dogs is not uncommon during acute, severe leptospiral infection. Vernel-Pauillac et al. [60] reported steady, gradual increases in IL-10 gene expression from 14 h after challenge to very high expression levels in hamsters 3 days after infection. Increased concentrations of IL-10 were also associated with fatal outcomes among hospitalized human patients with leptospiral infections [63], as seen in Study 1. The control dogs of Study 2 were asymptomatic, yet showed high levels of IL-10 PC, which may suggest that IL-10 has a protective effect in acute leptospirosis. This finding was also observed in human patients by Volz et al. [64]. It has been noted that there was a high IL10: TNF-α ratio in the control dogs experiencing acute leptospirosis, as was also reportedly found in human patients with fatal outcomes of acute leptospirosis [63]. The results obtained in the current investigation represent the first demonstration in dogs of a similar pattern of IL-10 and TNF-α, as previously observed in humans. Therefore, IL-10 might be protective against a severe course of infection or even the manifestation of symptoms.

There were no changes in IFN-γ and IL-4 concentrations measured, which were similar to the data obtained from a study that evaluated different markers found in severe leptospirosis using the hamster model [65], where no significant difference was observed in the gene expression levels of these two cytokines before and after leptospiral infection in the hamsters. Vernel-Pauillac et al. [60] hypothesized that high IL-10 levels measured in hamsters challenged with virulent leptospires regulated and limited the expression of IFN-γ. Similarly, in the dogs of both current studies, high IL-10 concentrations were also detected, which may have caused the regulated production of IFN-γ. Measuring gene expression by the hour would have enabled the detection of rapidly changing cytokine levels in whole blood samples using PCR amplification and analysis.

Our study observed no abnormalities in platelet levels following primary or secondary vaccinations, as all dogs remained clinically normal. Thrombocytopenia was the significant hematological abnormality observed in 9 (90%) of the 10 control dogs. This is considered a manifestation of a severe state of infection that resulted in septicaemia, and it is a common finding in human and animal leptospirosis, particularly when bleeding occurs [66]. All control dogs of Study 1 exhibited severe thrombocytopenia, which was consistent with the clinical signs of acute severe clinical leptospirosis. Most (80%) of the control dogs in Study 2 exhibited mild to moderate thrombocytopenia shortly PC, which persisted for a few days, and the dogs eventually recovered. It cannot be over-emphasized that none of the vaccinated dogs in Studies 1 and 2 exhibited thrombocytopenia. This was similarly found in other vaccination-challenge studies [44,53]. Hence, thrombocytopenia is a nonspecific indicator of acute leptospirosis in both symptomatic and asymptomatic infections.

Serum biochemistry analyses indicated that unvaccinated dogs had altered and impaired hepatic function, reflected by the concentrations of the enzyme ALT in the dogs that showed severe clinical disease (Study 1).

Bilirubin, another indicator of liver function, was also above the normal reference range in all controls (concentration range) that showed acute severe leptospirosis, compared to the concentration range found in vaccinated dogs. This is unsurprising because elevated bilirubin and icterus are manifestations of acute and severe canine leptospirosis. Similar findings have been documented in clinical canine leptospirosis [44,53,67].

Urea and creatinine levels were above the normal reference range, which indicates impaired kidney function. These findings were similarly reported for unvaccinated control dogs in a vaccine trial on leptospirosis by others [44,53]. Mechanism of their increase in canine leptospirosis and part of the indicators used in the panel for leptospirosis diagnosis [68].

Post-mortem findings in unvaccinated dogs challenged with virulent leptospires showed pathological changes consistent with leptospirosis, naturally or experimentally induced. These gross lesions (icterus, hemorrhages on the lungs, stomach, and intestines, hepatomegaly, and renal edema) were also consistent with the clinical signs of the dogs with leptospirosis, while alive. Similar post-mortem findings were reported in dogs that succumbed to the challenge by a virulent strain of leptospires [44,48,53,56]. The authors also documented no post-mortem lesions in dogs that survived the challenge, as found in the current investigation of vaccinated dogs in Studies 1 and 2 and unvaccinated control dogs in Study. It is difficult to induce clinical leptospirosis in adult dogs due to their more developed immune system, which will mount a rapid and effective response as seen in other vaccination-challenge studies [18,41,44].

Macroscopic findings at necropsy in the affected dogs were consistent with clinical signs and typical lesions of leptospirosis observed in dogs that succumbed to experimental challenge with virulent leptospires. Microscopic analysis of the vaccinated and unvaccinated dogs of Study 2 showed that vaccination protected against cellular changes to the examined viscera, since minor histological abnormalities were detected in vaccinated dogs. Changes observed in the unvaccinated group, the signs were consistent with mild to moderate leptospirosis, as reported in an earlier vaccine-challenge study [44,53], which included interstitial glomerulonephritis and tubular degeneration, as well as hepatic cellular dissociation. The cellular changes observed in pulmonary and intestinal tissue include lymphocytic and macrophagocytic infiltration of the lungs and expansion of interstitial tissue. However, others did not report lymphoid intestinal tissue [44,48,53,55,56].

Some limitations of our study include the following: (i) the small sample size of the dogs used in the study groups, (ii) the failure to assay for some cytokines such as IL-10, early, within hours post-challenge, since the serum levels decrease rapidly, and (iii) the use of culture only for detection of leptospires in urine to detect renal shedding, as qPCR was not available to the researchers at the time of the study

## 5. Conclusions

It is concluded that the killed whole-cell vaccine using serovar Copenhageni successfully protected vaccinated dogs from clinical disease and partially protected against chronic infection by reducing the frequency and duration of leptospiral urine shedding. Future work should include similar studies on serovar Mankarso, which is prevalent in dogs and rats in Trinidad and Tobago, and a higher number of beagle dogs in the study. It cannot be overemphasized that the vaccine produced from killed whole cells of a local strain of serovar Copenhageni has been demonstrated to be efficacious in hamsters, as reported by Suepaul et al. [40], and now in beagle dogs in the current study. Based on our findings, it can be inferred that IL-10 may be protective against a severe course of infection or the manifestation of symptoms. Additionally, a high IL-10 to TNF-α ratio, combined with thrombocytopenia, constitutes a non-specific marker of acute leptospirosis in symptomatic and asymptomatic infections.

It is recommended that based on the data obtained in the current which demonstrated the efficacy of the killed whole-cell serovar Copenhageni isolated locally in conjunction with the prevention of clinical leptospirosis and reduction in the frequency and durarion of renal shedding of leptospires in beagle dogs, the vaccine has the potential to be explored for production on a large scale for use in Trinidad and Tobago and possibly the Caribbean region at large. However, although a promising vaccine, it is prudent to conduct a larger study with a higher number of beagle dogs in each study group, and to concurrently compare its efficacy with that of the commercial leptospirosis vaccine currently available to prevent canine leptospirosis, as was done in the hamster model earlier.

## Figures and Tables

**Table 1:** Experimental design of the study.

Study	Type	Group	No. of Dogs	Challenge (Time After V2 *)
1	Onset of immunity	Control	5	2 weeks
		Vaccinated	5	
2	Duration of immunity	Control	5	14 months
		Vaccinated	7	

* V2 = Second vaccination/booster.

**Table 2:** Incidence and clinical severity of leptospirosis in vaccinated dogs and controls, after challenge with *Leptospira interrogans* serovar Copenhageni.

Study	Group	No. of Dogs	No. (%) with Clinical Disease, PC			
			None	Mild	Moderate	Severe ^a^
1	Controls	5	0 (0.0)	0 (0.0)	0 (0.0)	5 (100.0)
	Vaccinates	5	4 (80.0)	1 (20.0)	0 (0.0)	0 (0.0)
2	Controls	5	0 (0.0)	5 (100.0)	0 (0.0)	0 (0.0)
	6-week-old dog ^b^	1	0 (0.0)	0 (0.0)	0 (0.0)	1 (100.0)
	Vaccinates	7	7 (100.0)	0 (0.0)	0 (0.0)	0 (0.0)

^a^ The number of dogs exhibiting severe clinical disease is also the number of dogs that were euthanized after the challenge. ^b^ One 6-week-old beagle pup was added to Study 2 to demonstrate the virulence of the challenge organisms by inducing clinical signs of leptospirosis. A score of 0 = no disease, 1–2 = mild disease, 3–4 = moderate disease, and more than 4 = severe disease (indicated by red arrow).

## Data Availability

The original contributions presented in the study are included in the article/Supplementary Materials, further inquiries can be directed to the corresponding author.

## Supplementary Materials

The following supporting information can be downloaded at: https://www.mdpi.com/article/10.3390/pathogens14070611/s1, Figure S1: Mean IL-10 (±SE) concentrations for control and vaccinated dogs in Study 1; Figure S2: Mean IL-10 (±SE) concentrations for control and vaccinated dogs in Study 2; Figure S3: Mean white blood cell counts in dogs (vaccinated and unvaccinated controls) post-challenge in Studies 1 and 2; Figure S4: Mean ALT values in vaccinated and control dogs post-challenge in Studies 1 and 2.
