# Supplementary material for: Vaccination-Challenge Trials in Beagle Dogs Using Whole-Cell Leptospira interrogans Serovar Copenhageni Vaccine: Prevention of Clinical Leptospirosis, Serological, Leptospiremia, Leptospiruria, Cytokines, Hematological, and Pathological Changes
Source: Pathogens. 2025 Jun 20;14(7):611. doi: 10.3390/pathogens14070611 (PMC12300908; doi:10.3390/pathogens14070611)

Supplementary data: Figures S1, S2, S3 and S4

Figure S1. Mean IL-10 (±SE) concentrations for control and vaccinated dogs in Study 1

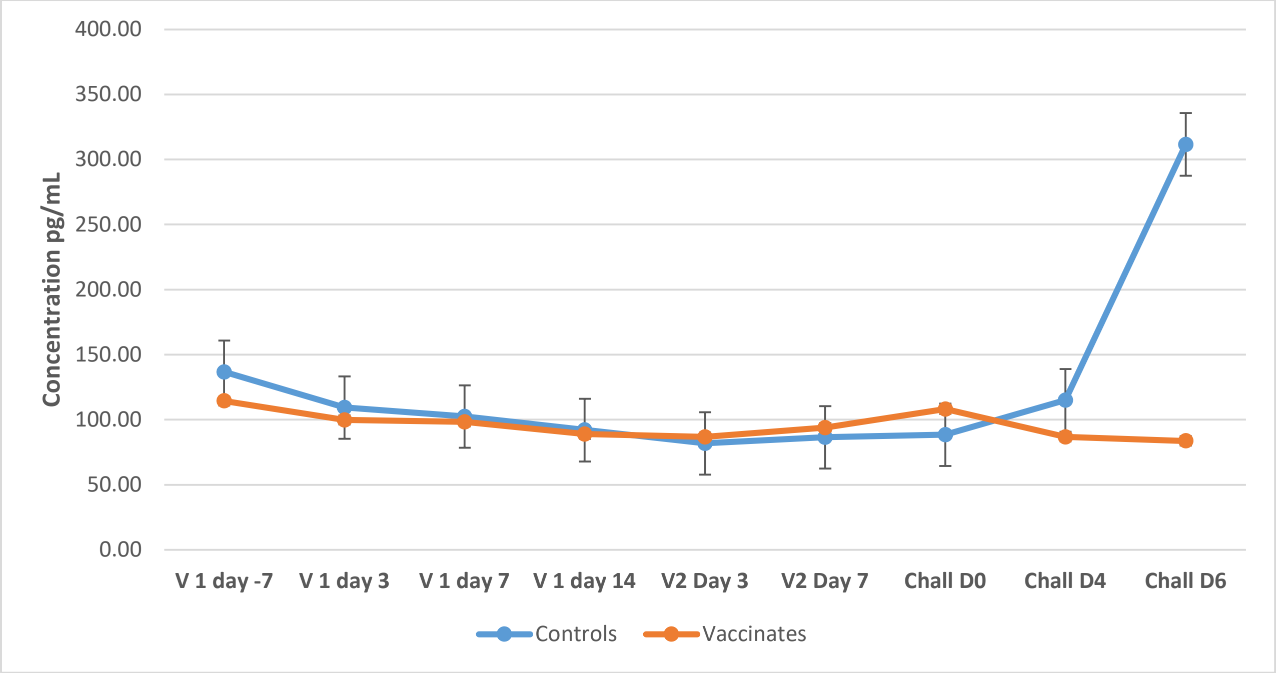

Figure S2. Mean IL-10 (±SE) concentrations for control and vaccinated dogs in Study 2

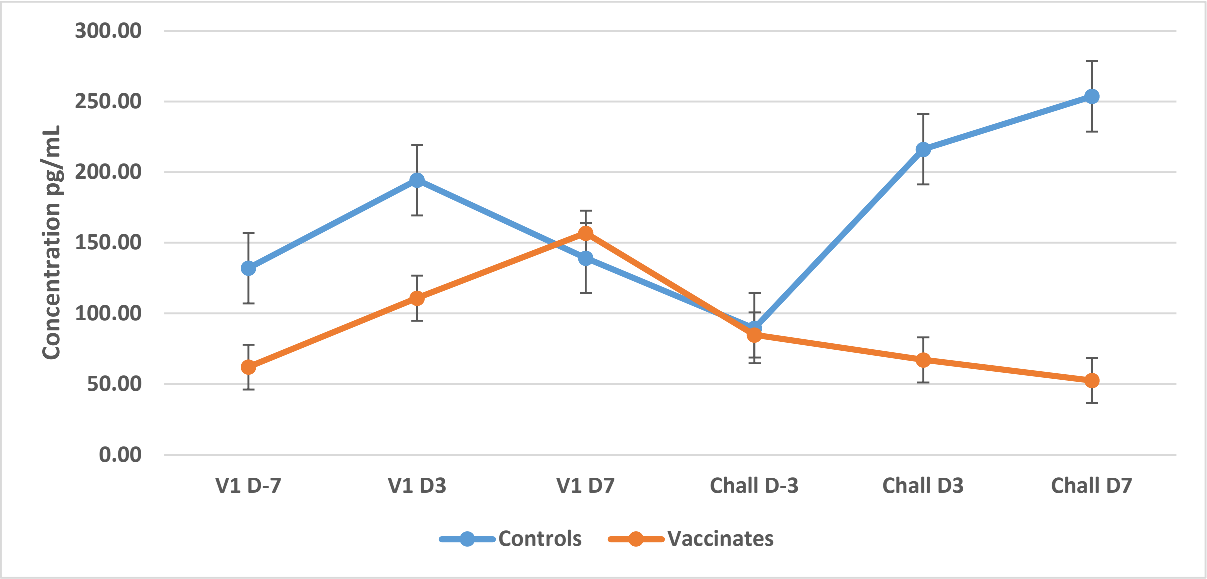

Figure S3. Mean white blood cell counts in dogs (vaccinated and unvaccinated controls) post-challenge in Studies 1 and 2

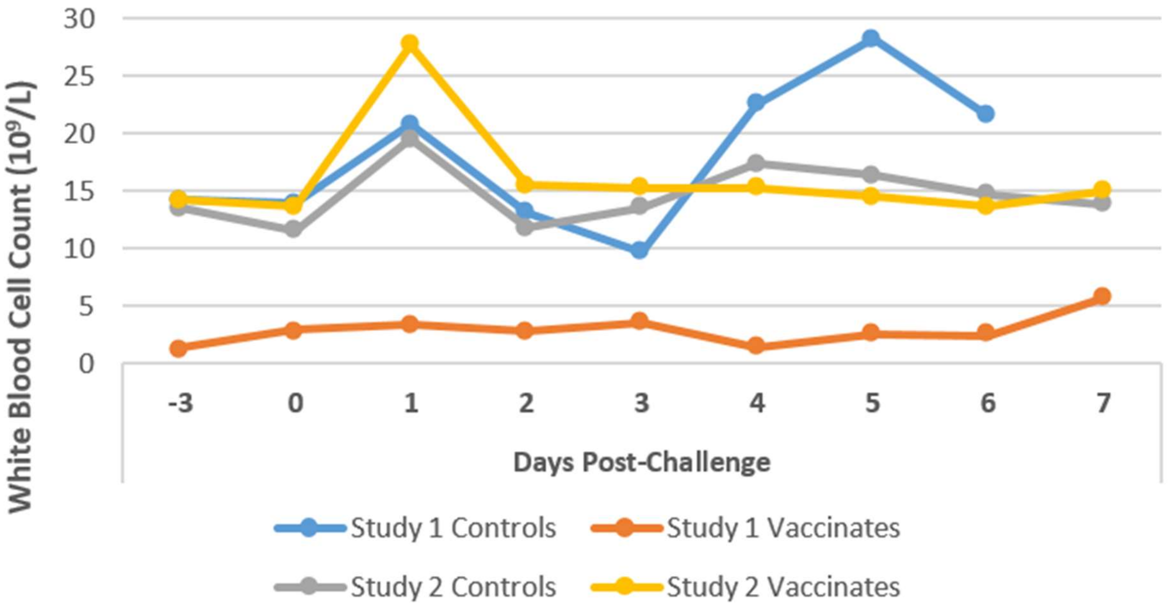

Figure S4: Mean ALT values in vaccinated and control dogs post-challenge in Studies 1 and 2

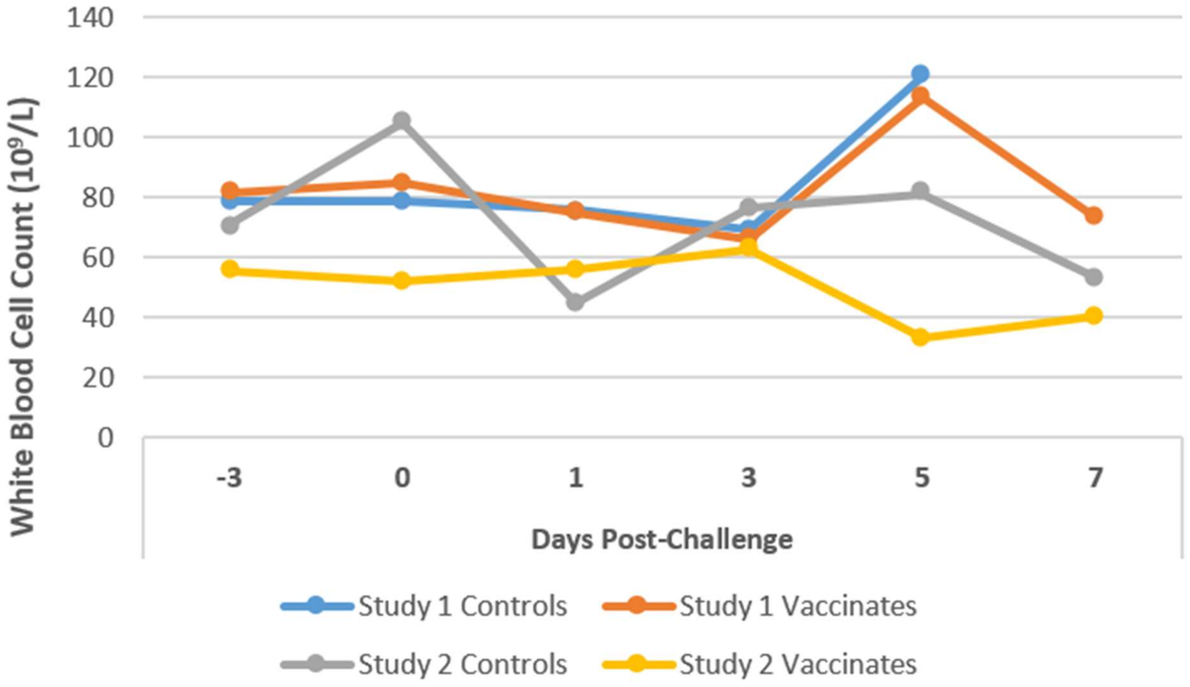

Supplement: Supplementary file 1 [file pathogens-14-00611-s001.zip › pathogens-3643514-supplementary.pdf]
